# miRNA-124 Prevents Rat Diabetic Retinopathy by Inhibiting the Microglial Inflammatory Response

**DOI:** 10.3390/ijms24032291

**Published:** 2023-01-24

**Authors:** Ying Chen, Andrea Schlotterer, Luke Kurowski, Lin Li, Marcus Dannehl, Hans-Peter Hammes, Jihong Lin

**Affiliations:** 1 5th Medical Department, Medical Faculty Mannheim, University of Heidelberg, D-68167 Mannheim, Germany; 2 Department of Vascular Surgery, Medical Faculty Mannheim, University of Heidelberg, D-68167 Mannheim, Germany; 3 Department of Pediatrics, Medical Faculty Mannheim, University of Heidelberg, D-68167 Mannheim, Germany

**Keywords:** miR-124, microglia, vasoregression, diabetic retinopathy

## Abstract

Diabetic retinopathy (DR) is characterized by vasoregression and glial activation. miRNA-124 (miR-124) reduces retinal microglial activation and alleviates vasoregression in a neurodegenerative rat model. Our aim was to determine whether miR-124 affects vascular and neural damage in the early diabetic retina. Diabetes was induced in 8-week-old *Wistar* rats by streptozotocin (STZ) injection. At 16 and 20 weeks, the diabetic rats were intravitreally injected with miR-124 mimic, and retinae were analyzed at 24 weeks. Microvascular damage was identified by evaluating pericyte loss and acellular capillary (AC) formation. Müller glial activation was assessed by glial fibrillary acidic protein (GFAP) immunofluorescence staining. Microglial activation was determined by immunofluorescent staining of ionized calcium-binding adaptor molecule 1 (Iba1) in whole mount retinae. The neuroretinal function was assessed by electroretinography. The expression of inflammation-associated genes was evaluated by qRT-PCR. A wound healing assay was performed to quantitate the mobility of microglial cells. The results showed that miR-124 treatment alleviated diabetic vasoregression by reducing AC formation and pericyte loss. miR-124 blunted Müller glial- and microglial activation in diabetic retinae and ameliorated neuroretinal function. The retinal expression of inflammatory factors including *Tnf-α*, *Il-1β*, *Cd74*, *Ccl2*, *Ccl3*, *Vcam1*, *Tgf-β1*, *Arg1*, and *Il-10* was reduced by miR-124 administration. The elevated mobility of microglia upon high glucose exposure was normalized by miR-124. The expression of the transcription factor PU.1 and lipid raft protein Flot1 was downregulated by miR-124. In rat DR, miR-124 prevents vasoregression and glial activation, improves neuroretinal function, and modulates microglial activation and inflammatory responses.

## 1. Introduction

Diabetic retinopathy (DR) is a microvascular complication of diabetes mellitus, and it is a major cause of blindness in diabetic patients worldwide [1]. DR affects the neuroglial compartment leading to neuronal dysfunction and neurodegeneration. Glial activation is a widely acknowledged hallmark in DR and contributes to its progression from the early non-proliferative to the proliferative stage [2]. Vasoregression is the primary lesion of DR and starts with pericyte dropout and AC formation [3]. Experimental evidence suggests that glial activation occurs prior to vascular damage [4,5,6].

In the healthy retina, microglia are quiescent and function as an immune watchdog to surveil the microenvironment. Once stimulated by low-level stress, for instance, infection or at the onset of diabetes, microglia become activated and produce pro- or anti-inflammatory cytokines in a balanced way to return the tissue to homeostasis [7]. However, as hyperglycemic damage accumulates, the delicate balance becomes impaired, and the harmful pro-inflammatory state becomes dominant through the release of excessive inflammatory molecules that ultimately result in neural damage [8,9]. This modulation of microglial activation in DR has been investigated in a few animal models [2,9,10]. For example, Zhang et al. demonstrated that the reduction of microglia-initiated inflammatory reactions alleviates diabetic retinopathy in a streptozotocin-induced diabetic mouse model [11]. Inhibiting the pathological activation of microglia or limiting its inflammatory response are proposed effective therapeutic approaches in retina degenerative disorders [2,8,12,13]. To prevent DR from developing to severe irreversible stages, investigations into new therapeutic approaches targeting microglia at earlier DR stages are needed.

miRNAs are small endogenous non-coding RNAs involved in many biological processes due to their role in post-transcriptional regulation. Dysregulated miRNAs are involved in the pathogenesis of certain diseases such as DR [14,15]. Approximately 350 miRNAs are expressed in a rat retina [16]. miR-124 is completely conserved and highly expressed in the central nervous system (CNS) [17,18,19,20]. Previously, we found that miR-124 distributes over the entire rat retina and colocalizes with Müller cell processes and the photoreceptor layers (PRL) [21]. miR-124 plays a pivotal role in the immune response in neurodegenerative diseases and inflammatory disorders, mainly through regulating microglia reactivity [22,23,24,25]. For example, the study in a mouse model of experimental autoimmune encephalomyelitis (EAE) demonstrated that overexpression of miR-124 promotes CNS microglial quiescence and suppresses EAE development, which is regulated by the transcriptional factor PU.1 [24]. The addition of miR-124 decreases microglial activation and inflammatory cytokine production, which contributes to spinal cord injury amelioration of the diseased rat model [23]. Our previous study revealed that the delivery of miR-124 into the retina reduces microglial activation and alleviates vasoregression in a neurodegenerative rat model [21]. However, whether miR-124 can improve the microvasculature and the microglia-associated inflammatory damage in experimental DR remains unclear. 

The aim of the present study was to clarify whether, under hyperglycemic conditions, miR-124 is able to alleviate vasoregression and microglial activation and preserve neuroretinal function.

## 2. Results

### 2.1. miR-124 Alleviates Diabetic Retinal Vasoregression

**Figure 1:**
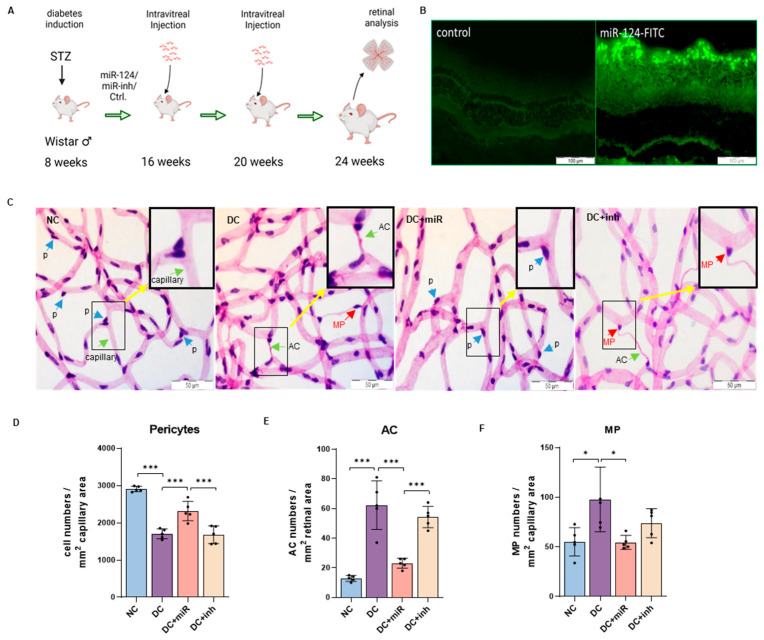
Effects of miR-124 on pericyte loss and AC formation in diabetic rat retinae. (**A**) Schema of animal experiments. According to the diabetes-induction and miR-124 injection, the animals are divided into four groups: non-diabetic control (NC), diabetes (DC), diabetes treated with miR-124 (DC+miR), or diabetes treated with miR-124 inhibitor (DC+inh). (**B**) Images of green fluorescence from the eyes injected with 25 pmol of miR-124-FITC (right) or with solvent as control (left) after 3 h. Images were taken with Olympus BX51 microscope, scale bar = 100 µm. (**C**) Representative images of PAS and hematoxylin-stained retinal digestion preparation of rats from control and experimental groups. Images were taken with Olympus BX51 microscope. AC: acellular capillary, MP: migrating pericyte, p: pericyte; scale bar = 50 µm. (**D**–**F**) Quantification of pericytes (number of pericytes/mm^2^ capillary area) (**D**); of acellular capillaries (number of AC/mm^2^ retinal area) (**E**); of migrating pericytes (number of MP/mm^2^ capillary area) (**F**) were analyzed using Cell^F^ software version 5.1 from Olympus. *n* = 5, * *p* < 0.05, *** *p* < 0.001 (one-way ANOVA with Tukey’s multiple comparisons test).

As described in the schema in Figure 1A, we obtained the STZ-induced diabetic *Wistar* rats and divided the experimental animals into four groups: non-diabetic control (NC), diabetes (DC), diabetes treated with miR-124 (DC+miR), and diabetes treated with miR-124 inhibitor (DC+inh). The efficiency of intravitreal miRNA introduction was confirmed by monitoring the green fluorescence of miR-124-FITC mimic in the rat eyes 3 h after injection (Figure 1B). To evaluate the influences of miR-124 in the vasculature of the diabetic retina, we analyzed the retinal digest preparations from the four groups for the development of pericyte loss and the formation of ACs by retinal morphometry (Figure 1C). As expected, in the DC retinae, the number of pericytes was reduced by 42% (Figure 1D), the number of ACs was increased by 355% (Figure 1E), and the number of migrating pericytes (MP) was increased by 80% (Figure 1F) in comparison to the data in the NC group. The introduction of exogenous miR-124 in DC animals (DC+miR) prevented the loss of pericytes by 24% compared to the DC control group (*p* < 0.001) (Figure 1D). Likewise, exogenous miR-124 reduced the formation of AC by 63% (*p* < 0.001) (Figure 1E) and the number of MP by 48% (*p* < 0.05) (Figure 1F) in comparison to the diabetic group (DC+miR vs. DC). The inhibitor of miR-124 did not improve the vascular pathology of diabetic retinae (Figure 1C, DC+inh vs. DC; Figure 1D–F). These results indicated that miR-124 prevented vasoregression in diabetic retinae.

### 2.2. miR-124 Reduces Müller Glial Activation in the Diabetic Retina

**Figure 2:**
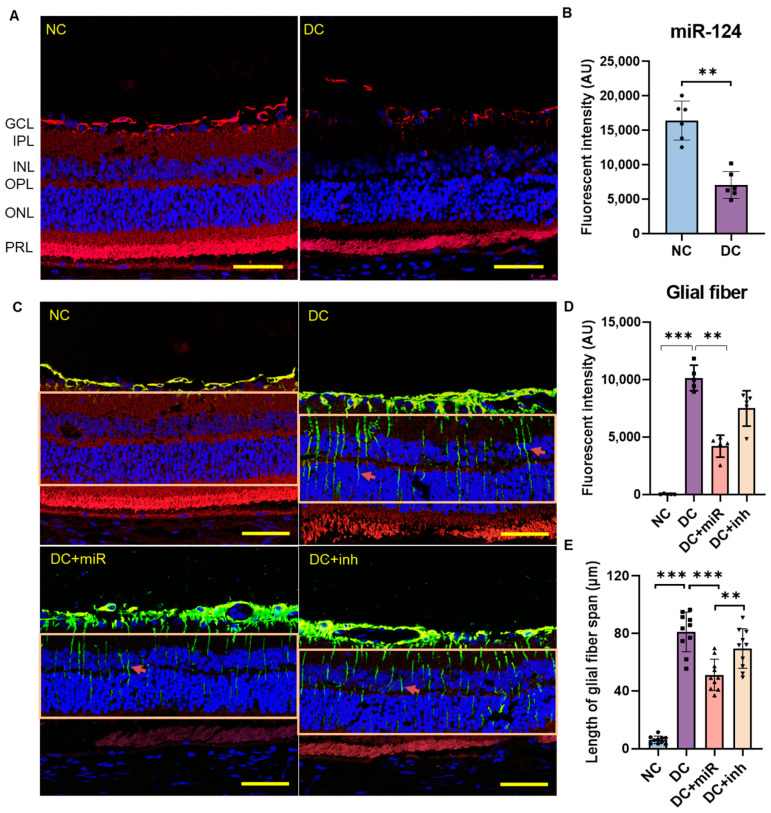
The expression of miR-124 in rat retinae and its effect on the activation of Müller glia in diabetic retinae. (**A**) Representative images of ISH probed with miR-124 (red) on the paraffin-embedded retinal vertical sections of 24-week-old healthy *Wistar* rats (NC) and diabetic *Wistar* rats (DC). GCL: ganglion cell layer; IPL: inner plexiform layer; INL: inner nuclear layer; OPL: outer plexiform layer; ONL: outer nuclear layer; PRL: photoreceptor layer. (**B**) Quantification of miR-124 ISH fluorescent intensity in (**A**) using Image J/Fiji software version 1.53c. Data are exhibited as mean ± SD, *n* = 6, ** *p* < 0.01 (*t*-test). (**C**) Representative images of ISH probed with miR-124 in combination with immunofluorescence staining of GFAP performed on the retinae of non-diabetic control rats (NC), diabetic rats (DC), DC treated with miR-124 (DC+miR), or DC treated with miR-124 inhibitor (DC+inh). The miR-124 probe was labeled with Alexa Fluor 555 (red), GFAP was labeled with Alexa Fluor 488 (green), and nuclei were labeled with DRAQ5^TM^ (blue). (**D**) Quantification of GFAP fluorescence in Müller glial radial fibers (indicated with arrows) within marked rectangular areas was performed using Image J/Fiji software. (**E**) Quantification of the length of endfeet of Müller glia down to the deep retinal layer performed using Image J/Fiji software. Data are exhibited as mean ± SD, *n* = 5–10, ** *p* < 0.01, *** *p* < 0.001 (one-way ANOVA with Tukey’s multiple comparisons test). The images were taken with the Leica confocal microscope SP8, scale bar = 50 µm.

To detect the expression of miR-124, we performed ISH on the vertical sections of the retinae of control and diabetic *Wistar* rats. In healthy retinae, miR-124 was detected in the entire layers of retinal neurons, including the ganglion cell layer (GCL), inner plexiform layer (IPL), inner nuclear layer (INL), outer plexiform layer (OPL), outer nuclear layer (ONL), and mostly in the photoreceptor layer (PRL) (Figure 2A, NC). However, in diabetic retinae, miR-124 expression was markedly decreased (57% reduction, DC vs. NC, *p* < 0.01, Figure 2A,B).

Glial activation is well appreciated in DR. To determine the effect of miR-124 on glial activation in the diabetic retina, ISH of miR-124 combined with immunofluorescent staining of GFAP was performed. GFAP was moderately expressed in astrocytes in the GCL of non-diabetic control retinae (Figure 2C, NC), whereas, in diabetic retinae, it was expressed more strongly in astrocytes in GCL and newly detected in Müller glia radial fibers (Figure 2C, DC). The enhanced fluorescent intensity of GFAP in Müller glia fibers (Figure 2D) and the increased length of the endfeet of Müller glia down to the deep retinal layer (Figure 2E) indicate the strong activation of Müller glia in diabetic retinae. Delivery of miR-124 into diabetic eyes left GFAP levels in astrocytes unchanged. In contrast, the fluorescent signals in Müller glia fibers were significantly inhibited (58% reduction, DC+miR vs. DC, *p* < 0.01, Figure 2C,D), and the length of the endfeet down to the deep capillary layer was significantly decreased (37% reduction, DC+miR vs. DC, *p* < 0.001, Figure 2C,E). The diabetic rats injected with the miR-124 inhibitor (miR-inh) did not show an inhibitory effect on GFAP expression. Altogether, these results indicate that miR-124 is able to reduce Müller glial activation under hyperglycemic conditions.

### 2.3. miR-124 Reverses Microglial Activation in the Deep Layer Retina of the Diabetic Rat

**Figure 3:**
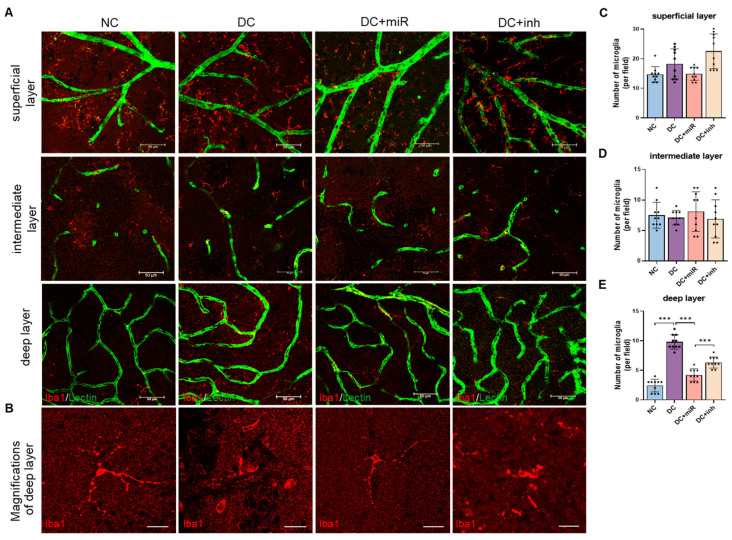
miR-124 normalized deep layer microglial activation in diabetic retinae. (**A**) Representative images of co-immunofluorescent staining of Iba1 (red) and Lectin (green) in the whole mount retinae from non-diabetic control (NC) rats, diabetic rats (DC), DC treated with miR-124 (DC+miR), or DC treated with miR-124 inhibitor (DC+inh) in superficial, intermediate, and deep vascular layer. The images were taken with the Leica confocal microscope SP8, scale bar = 50 µm. (**B**) The representative images of magnifications of Iba1 staining in the deep layer. Scale bar = 20 µm. (**C**–**E**) Quantification of microglia in the superficial layers (**C**), intermediate layers (**D**), and deep layers (**E**) of rat retinae. The quantification of microglial numbers was exhibited as mean ± SD, *n* = 10, *** *p* < 0.001 (one-way ANOVA with Tukey’s multiple comparisons test).

Microglia become activated in diabetes. To assess whether miR-124 had an effect on microglial activation, we performed immunostaining in whole mount retinae for Iba1. The Iba1-positive cells were quantified from superficial, intermediate, and deep retinal vascular layers. As expected, activated microglia were recruited to diabetic retinae (DC) compared to the normal control retinae (NC) in both the superficial layer (Figure 3A upper panel and Figure 3C) and the deep layer (Figure 3A lower panel and Figure 3E, *p* < 0.001). In comparison to the diabetic retinae without injection (DC) or the diabetic retinae with miR-inh injection (DC+inh) control groups, the application of miR-124 into diabetic rats (DC+miR) reduced the levels of microglial activation predominantly in the deep layer (38% reduction, DC+miR vs. DC, *p* < 0.001, Figure 3A lower panel and Figure 3E). The morphology of microglia in diabetes was changed from a small cell body with very fine and highly ramified processes (Figure 3B, NC) to an activated form with a bigger cell body and shorter processes (Figure 3B, DC). With the replacement of miR-124, the activated form was returned to a resident state similar to NC. No significant differences between groups were observed in the intermediate retinal layer (Figure 3A middle panel and Figure 3D).

### 2.4. miR-124 Preserves Neuroretinal Function in Diabetic Rats

**Figure 4:**
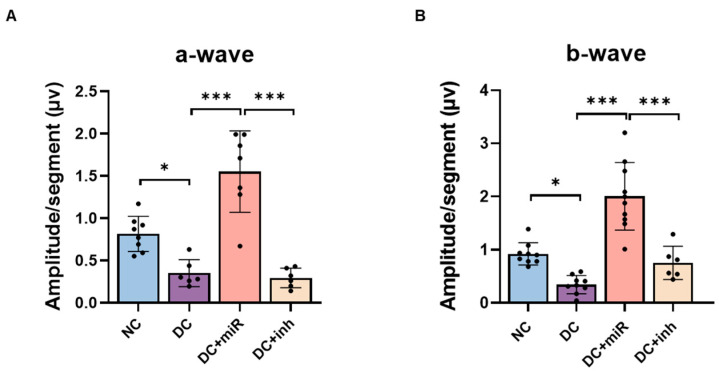
Effects of miR-124 on neuroretinal functions of diabetic rats. (**A**) Averaged values of a-wave amplitudes and (**B**) b-wave amplitudes measured by mfERG on rats at week 24 in four experimental groups: non-diabetic control (NC), diabetic control (DC), diabetic rats treated with miR-124 (DC+miR) or with its inhibitor (DC+inh). Data are presented as mean ± SD, *n* = 6–8. *p* values were determined by one-way ANOVA with Tukey’s multiple comparisons test, * *p* < 0.05, *** *p* < 0.001.

To evaluate the effect of miR-124 on retinal function, we measured the ocular responses to light stimulation by multifocal electroretinography (mfERG). In diabetic retinae, the amplitudes of the a-wave, representing the function of photoreceptors (Figure 4A), and the b-wave, representing the function of bipolar and Müller cells (Figure 4B), were significantly reduced in comparison to NC retinae, implying that the functions of both photoreceptor and Müller glia were impaired in diabetes. The delivery of miR-124 into diabetic retinae improved the light reactions for both waves. These mfERG results imply that miR-124 could prevent damage of hyperglycemia on photoreceptors and Müller glia to rescue neuroretinal functions.

### 2.5. miR-124 Suppresses the Inflammatory Responses of Reactivated Microglia in Diabetic Retinae

**Figure 5:**
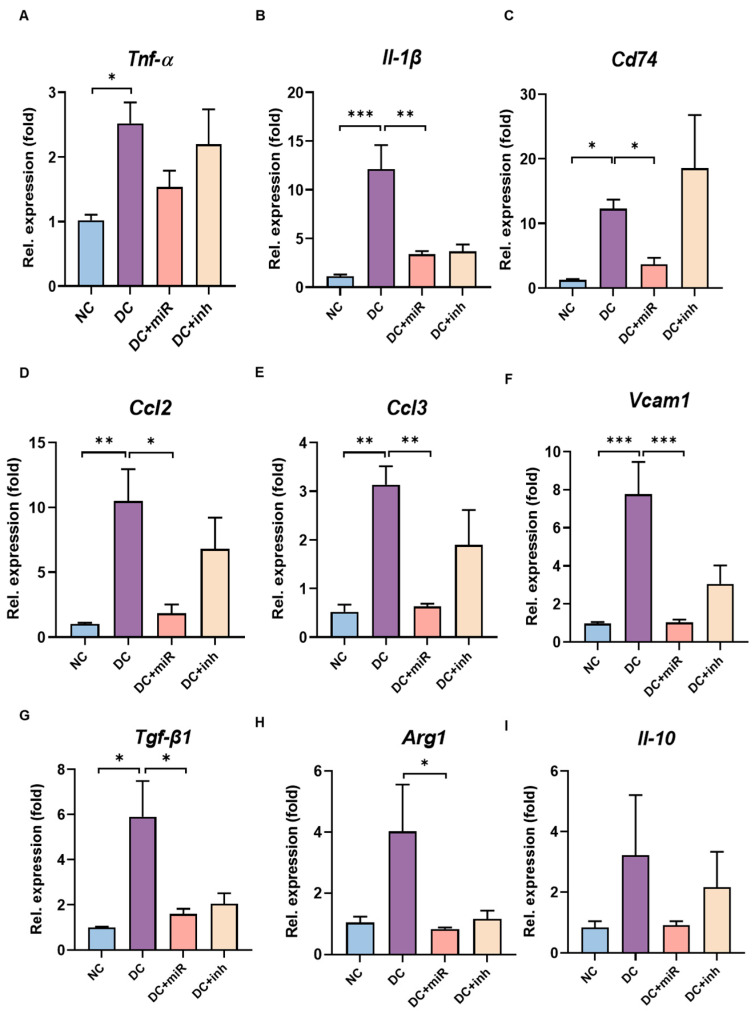
miR-124 inhibited the inflammatory molecules in diabetic retinae. The relative expression of inflammatory-associated genes evaluated by quantitative RT-PCR in the samples of rat retinae from groups of non-diabetic control (NC), diabetic control (DC), DC treated with miR-124 (DC+miR) or its inhibitor (DC+inh). (**A**) *Tnf-α*, (**B**) *Il-1β*, (**C**) *Cd74*, (**D**) *Ccl2*, (**E**) *Ccl3*, (**F**) *Vcam1*, (**G**) *Tgf-β1*, (**H**) *Arg1*, and (**I**) *Il-10*. The expression of the housekeeping gene rat *Gapdh* was used as a control. Relative gene expression (fold versus *Gapdh*) was calculated using ΔΔ*CT* method. Data are shown as mean ± SD, *n* = 5, * *p* < 0.05, ** *p* < 0.01, *** *p* < 0.001 (one-way ANOVA with Tukey’s multiple comparisons test).

To assess the effect of miR-124 on microglia inflammatory response in persistent hyperglycemic conditions, we examined the expression of inflammation-associated genes in the retinae of NC, DC, DC+miR, and DC+inh rats using quantitative RT-PCR. In contrast to the expression in NC, a large range of inflammatory molecules were notably enhanced in DC retinae, including cytokines (*Tnf-α*, *Il-1β*), CD74 molecule (*Cd74*), chemokines (*Ccl2*, *Ccl3*), vascular cell adhesion protein 1 (*Vcam1*), *Tgf-β1*, *Arg1*, and *Il-10* (Figure 5). Importantly, the elevated expression of these genes was specifically inhibited by the introduction of miR-124, which demonstrates the inhibitory effects of miR-124 in the inflammatory responses of microglia in diabetic conditions.

### 2.6. miR-124 Inhibits the Mobility of Microglial Cells in High Glucose Conditions

**Figure 6:**
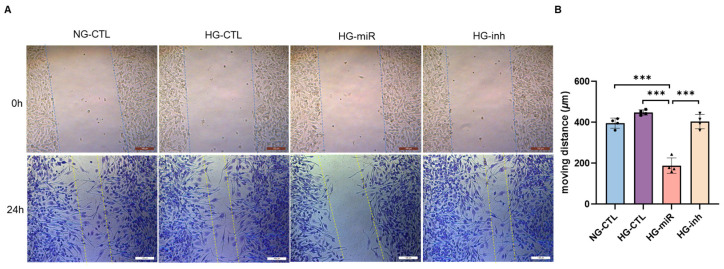
Effect of miR-124 on microglia mobility in high glucose conditions. (**A**) Representative images of WHA performed with high glucose (4.5 g/L)-treated BV2 cells which were transfected with control miRNA (HG-CTL), or miR-124 mimic (HG-miR) or miR-124 inhibitor (HG-inh) for 24 h. Cells cultured in normal glucose medium and transfected with control miRNA were used as control (NG-CTL). At the endpoint, cells were fixed in methanol and stained with Giemsa. The images at the time point of 0 h (upper panel) and 24 h (low panel) were taken with the Leica DMIRB light microscope, scale bar = 200 µm. (**B**) Quantification of the moving distances calculated from the gap sizes measured at 0 h and 24 h, 10 random measurements of each image, 4 images from each group. Data are shown as mean ± SD, *n* = 4, *** *p* < 0.001 (one-way ANOVA with Tukey’s multiple comparisons test).

As migration is a key feature of microglial activation and its immunological surveillance function, a wound healing assay (WHA) was carried out to evaluate the effect of miR-124 on the mobility of BV2 microglial cells in high glucose conditions (HG). The results showed that the moving speed—negatively reflected by the decrease in gap size—of miR-124 transfected BV2 cells (HG-miR) was significantly slower than those in the control miRNA transfected group (HG-CTL) (58% reduction, *p* < 0.001) or in the miR-124 inhibitor transfected group (HG-inh) (48% reduction, *p* < 0.001) (Figure 6A,B). These WHA results illustrate that miR-124 is able to limit the mobility of retinal microglia in diabetic conditions. As shown in a previous study [21], the 24 h transfection efficiency of miR-124, control miRNA, or miRNA inhibitor into BV2 cells was similar.

### 2.7. PU.1 and Flot1 Are Down-Regulated by miR-124 Introduction

**Figure 7:**
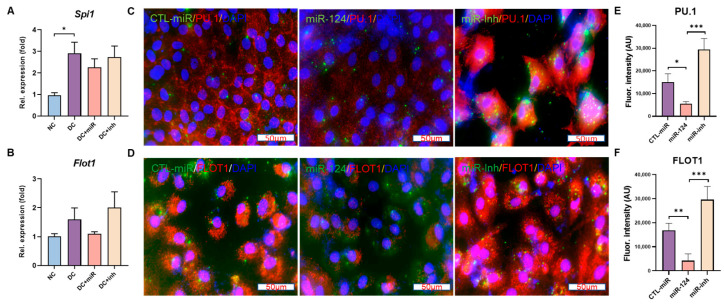
Downregulation of PU.1 and Flot1 by miR-124. (**A**,**B**) The relative expression of gene Spi1 (**A**) and Flot1 (**B**) in the rat retinae of non-diabetic controls (NC), diabetic controls (DC), DC treated with miR-124 (DC+miR) or its inhibitor (DC+inh) measured by quantitative RT-PCR. (**C**,**D**) Immunocytochemistry (ICC) of PU.1 (**C**) and FLOT1 (**D**) in primary rat microglial cells transfected with control miRNA (CTL-miR), miR-124 mimic (miR-124), or miR-124 inhibitor (miR-inh). PU.1 and FLOT1 were detected using Alexa Fluor 555 labeled secondary antibody (red), miRNA localization was visualized by FITC (green) due to its fluorescein-labeled form, and cell nuclei were labeled with DRAQ5^TM^ (blue). Images were taken with the Leica confocal microscope SP8, scale bar = 50 µm. (**E**,**F**) Quantification of ICC immunofluorescence of PU.1 (**E**) and FLOT1 (**F**) using Image J/Fiji software. Data are exhibited as mean ± SD, *n* = 5, * *p* < 0.05, ** *p* < 0.01, *** *p* < 0.001 (one-way ANOVA with Tukey’s multiple comparisons test).

Given that transcription factor PU.1 and lipid raft protein Flot1, which are indirect or direct targets of miR-124, are involved in the miR-124 regulation of microglial behaviors in neurodegenerative disease models [21,24], we investigated if similar regulatory mechanisms are valid in diabetes. The expression of PU.1 and Flot1 was evaluated both in vivo and in vitro. The results showed that the mRNA levels of *Spi1* (encoding PU.1) and *Flot1* were upregulated in diabetic rat retinae compared to those in normal retinae (Figure 7A,B). After 8 weeks of supplementation of miR-124 into the diabetic eyes (DC+miR), both genes were downregulated by 22% compared to non-treated diabetic groups (DC). The downregulation of both proteins was also observed in primary rat microglial cells transfected with miR-124 (63% reduction for PU.1, *p* < 0.05 and 75% reduction for FLOT1, *p* < 0.01, Figure 7C–F) in comparison to that in control miRNA transfected cells. Together, our results indicate that PU.1 and Flot1 are involved in miR-124-regulated microglial activation and inflammatory responses under hyperglycemic conditions.

## 3. Discussion

In the present study, we investigated the role of miR-124 in regulating microglia and in the progression of rat DR. Our results showed that Müller glia and microglia were over-activated in the diabetic rat retina and were returned to the non-diabetic control state with the introduction of miR-124. Regarding the vascular changes, the number of pericytes dropped, and ACs increased in diabetic rat retinae, which were normalized upon the administration of miR-124. The neuroretinal function was impaired in rats 4 months after diabetes onset. This functional impairment was reversed in miR-124 treated retinae for both photoreceptor and Müller glia/bipolar cell function. The inflammatory responses of microglia were enhanced in diabetic retinae and considerably inhibited by miR-124. The excessive movement of microglial cells in high glucose conditions was restrained by miR-124 treatment. Hence, miR-124 administration is a promising strategy for regulating microglial activation and immune response during early DR. 

Müller glia undergoes reactivation and dysfunction in pathological conditions, for example, in the ciliopathy neurodegenerative model, where microglial activation is associated with retinal vasoregression [26]. Our previous study has further identified that miR-124 replacement reduces gliosis and rescues the neuroretinal functions of Müller glia in this neurodegenerative rat model and hypothesized that microglia mediates the miR-124 regulation on vasoregression and Müller glial function [21]. In the present study under hyperglycemic conditions, we observed similar effects that miR-124 replacement reduces microglial and Müller glial activation, alleviates vasoregression, and preserves neuronal function. We identified that the distribution of miR-124 is mainly in the Müller cells and PRL in the rat retina. In healthy conditions, Müller glia mediates the signal transduction and molecule transport between neurons and vascular cells [27,28] and regulates microglial dynamic motility [29]. We deduce that the protective function of miR-124 is mainly accomplished through Müller cells. Under pathological conditions, e.g., hyperglycemia, the level of miR-124 in Müller cells decreases, and Müller cells undergo gliosis and dysfunction. After the replacement of miR-124 in the diabetic retinae, glial activation was inhibited, and vascular and neuroretinal function was recovered. We speculated that the elementary downstream effector of miR-124 treatment was microglia since they are the immune cells in the retina and execute surveillance functions. The exogenous miR-124 induced microglial deactivation and dampened the inflammatory responses. The beneficial signals were transferred from microglia to Müller glia [29], subsequently transmitted to neurons and vascular cells, and achieved neurovascular unit (NVU) protection. Therefore, miR-124 provides neuroprotection in diabetes via regulating microglial and Müller glial activation.

Both in patient and animal models, the detrimental influence of hyperglycemia on the inner retinal neuronal viability and function is described. Yet, the impact of hyperglycemia on photoreceptor function remains controversial [30,31,32,33]. In our study, we noted the impaired functions of Müller glia/bipolar cells and photoreceptors in 4-month diabetic rats, which is consistent with the observations of others [34,35,36,37]. Interestingly, the delivery of miR-124 mimics into the eyes of a photo-oxidative damage (PD) mouse model reduces inflammation and photoreceptor loss and preserves retinal function. [38]. These observations and the results of our study reinforce the beneficial role of miR-124 in neuroretinal disorders. Together, we postulate that the introduction of miR-124 in the early diabetic retina prevents functional impairment of Müller glia and photoreceptors due to hyperglycemia.

In this study, we identified that diabetes significantly upregulated a large range of inflammatory mediators in rat retinae, including cytokines (*TNF-α*, *IL-1β*, *CD74*, *TGF-β1*, *IL-10*), chemokines (*CCL2*, *CCL3*), adhesion molecules (*VCAM1*), and arginase 1 (*Arg1*), all of which were returned almost to normal control levels with the supplementation of miR-124. We speculated that, in diabetic rats, persistent tissue stress (hyperglycemia) directs over-activated retinal microglia into a potential irreversible pro-inflammatory state, releases excessive pro-inflammatory molecules, aggravates inflammation, and ultimately results in retinal vascular damage. This deleterious inflammatory reaction could be prevented by miR-124 treatment at earlier stages. 

Migration is a key feature of microglial activation in response to neuronal injury or inflammatory stimuli in the CNS [39,40]. The result observed in BV2 microglial cells reinforces our previous observation in the rat primary microglial cells [21] that miR-124 inhibits the mobility of microglia also under diabetic conditions. Therefore, miR-124 reduces microglial activation both in neurodegeneration and inflammation-associated retinopathy.

To elucidate the potential mechanisms involved in the miR-124 regulation of microglial behavior in early diabetes, we detected the expression of PU.1 and Flot1 in diabetic rat retinas and rat primary microglial cells with miR-124 treatment. PU.1—together with its upstream regulator C/EBP-α, which is a direct target of miR-124—is involved in the regulation of microglia in the retina and CNS. PU.1 has been associated with microglial activation in a neurodegenerative mouse model [41]. Silencing PU.1 with siRNA inhibited the viability and phagocytic function of brain microglia [42]. Our previous study demonstrated that with the addition of miR-124, the expression of PU.1 is downregulated, and the microglial activation is inhibited in the neurodegenerative rat retina [21]. In the present study, PU.1 levels were downregulated in diabetes upon the miR-124 introduction. This indicates that, in both neurodegenerative diseases and under diabetic conditions, PU.1 is an important mediator of retinal microglia regulation. 

Similarly, we observed that Flot1, a direct binding target of miR-124, was suppressed in diabetic retinae and the rat primary microglial cells after miR-124 treatment. Flot1 is a key lipid raft protein and plays an important role in vesicular trafficking and signal transduction [43,44]. Abnormal lipid raft formation hampers signal transduction, for example, in the TNF-α induced inflammatory response of microglia [21]. Therefore, PU.1 and Flot1 are obvious effectors of miR-124-mediated microglial function not only in neurodegeneration but also in diabetes. The precise mechanisms involved deserve further study.

In conclusion, miR-124 prevents vasoregression and Müller glial/microglial activation in early diabetes. miR-124 protects photoreceptors and Müller glia damage from hyperglycemia and thus rescues neuroretinal function and guarantees the normal and effective function of the neurovascular unit. miR-124 inhibits microglial activation in diabetes by reducing their immune responses and mobility. Therefore, miR-124 represents a promising mediator of vasculature maintenance and avoiding microglial over-activation in early diabetes. 

Despite the small size and non-coding properties, as well as the vascular and neural protective quality, miR-124 is a promising approach to prevent the progression of incipient DR. Clearly, intravitreal injection into the eyes of patients with mild to moderate non-proliferative diabetic retinopathy (NPDR) is clinically inappropriate at present. Further studies on the approaches of application, for example, via eye drops, will help in assessing its therapeutic potential.

## 4. Materials and Methods

### 4.1. Animal Experiments

Total of 60 eight-week-old male *Wistar* rats (albino) were purchased from Charles River and kept in a 12 h light/dark cycle with ad libitum access to food. Animals were randomly divided into four groups (15 rats per group). All animals fasted overnight. Three groups of animals were induced diabetes by a single intraperitoneal injection of a freshly prepared streptozotocin solution (STZ, dissolved in 0.05 M citrate buffer, pH 4.5; S0130, Sigma-Aldrich Chemie GmbH, Schnelldorf, Germany) at a dose of 35 mg/kg body weight. One group of animals injected with only citrate buffer served as non-diabetic control (NC). The blood sugar and body weight of diabetic animals were monitored weekly with a glucometer strip (BG Star^®^, Sanofi-Aventis, Frankfurt am Main, Germany) and a digital balance. Hyperglycemia was confirmed by blood glucose levels higher than 250 mg/dL. Rats with high blood sugar (>600 mg/dL) were given 2 units of insulin (Lantus^®^ Solostar^®^ Insulin glARGin 100 unit/mL, Sanofi-Aventis) 3 times per week subcutaneously. Diabetic rats (DC) at week 16 and week 20 were intravitreally injected with 25 pmol of miR-124 mimic (471256-001, Qiagen, Hilden, Germany, sequence: 5′-UAAGGCACGCGGUGAAUGCC-3′) (DC+miR), or miR-124 inhibitor (4102198-001, Qiagen, sequence 5′-GCATTCACCGCGTGCCTTA-3′) (DC+inh). The diabetic rats injected with only solvent were used as controls (DC). Experimental rats were euthanized at week 24, and the eyes were enucleated and frozen at −80 °C. *Wistar* rats were intravitreally injected with 25 pmol of FITC labeled miR-124 mimic (479995-011) or solvent. The eyes were enucleated 3 h after injection and sliced into 6 µM cryosection. The green fluorescence was detected with Olympus BX51 microscope. The animal experiments were approved by the local authorities (animal license numbers G-150/16, Regierungspräsidium, Karlsruhe, Germany) and carried out in compliance with the statement of Association for Research in Vision and Ophthalmology (ARVO). 

### 4.2. Retinal Digestion Preparation and Morphometry

The retina was dissected from the rat eye and digested with 3% trypsin (Porcine Trypsin, 85450C, Merck KGaA, Darmstadt, Germany) to obtain the retinal vascular preparation as described previously [45,46]. The vascular net was stained with Periodic Acid Schiff’s (PAS) reagent and hematoxylin, then photographed with an Olympus BX51 Microscope. The quantitative retinal morphometry was analyzed with the Cell^F^ system version 5.1 (Olympus Opticals, Hamburg, Germany). Briefly, the quantification of pericytes or migrating pericytes (MP) was calculated as cell numbers relative to the retinal capillary area (cell number/mm^2^ capillary area); the quantification of AC was calculated as numbers relative to the retinal area (AC number/mm^2^ retina area) according to the established methods and morphology definition described previously [47,48,49].

### 4.3. In Situ Hybridization (ISH) and Fluorescent Immunohistochemistry

ISH combined with immunofluorescent staining was performed to detect miR-124 expression and glial activation as described previously [21]. Briefly, 6 µm thickness tissue sections from formalin-fixed and paraffin-embedded retinae were hybridized with 80 nM digoxigenin (DIG) labeled miRCURY LNA miR-124 Detection Probe (Qiagen) or scrambled miRNA probes (Qiagen) at 53 °C for 1 h. After washing, the sections were incubated with sheep anti-digoxigenin (11333089001, Roche, 1:800) and goat anti-GFAP (sc-6170, Santa Cruz Biotechnology, Heidelberg, Germany, 1:100) antibodies at 4 °C overnight. The secondary antibodies of donkey anti-sheep Alexa Fluor 555 (A21436, Invitrogen, Schwerte, Germany, 1:200) and chicken anti-goat Alexa Fluor 488 (21467, Invitrogen, 1:200) were incubated at room temperature for one hour. The nuclei were stained with DRAQ5^TM^ (65-0880, Invitrogen, 1:1000) for 10 min. Images were acquired with the Leica TCS SP8 confocal microscope (Leica Microsystems, Wetzlar, Germany) under constant exposure time and gain for all specimens. The quantification of GFAP fluorescence intensity in Müller glial radial fibers and the measurement of the length of the endfeet of Müller glia were performed using Image J/Fiji software version 1.53c.

### 4.4. Retinal Whole Mount Immunofluorescent/Histochemical Staining

After a rat eye was fixed in 4% paraformaldehyde (PFA) overnight, the whole mount retina was dissected. The retina was blocked and permeabilized in 1% BSA and 0.5% Triton X-100 for 2 h, then labeled with FITC conjugated lectin (L9381, Sigma-Aldrich, 1:100) at room temperature for 7 h, followed by incubation with anti-Iba1 antibody (019-19741, Wako Chem, Neuss, Germany, 1:100) at 4 °C overnight. The retina was then incubated with secondary antibody Alexa Fluor 555 donkey anti-rabbit (A31572, Invitrogen, 1:200) at room temperature for one hour. All images were scanned using the Leica TCS SP8 confocal microscope. Iba1-expressing cells were quantified in ten randomly selected fields (400× magnification) from superficial, intermediate, and deep vascular retinal layers.

### 4.5. Multifocal Electroretinography (mfERG)

The neuroretinal function was detected by multifocal electroretinography (mfERG) under photopic conditions as previously described [50,51]. Rats were anesthetized using Isofluran (1ml/mL, CP-pharma, Burgdorf, Germany). RETImap system was used for the measurement (Roland Consult, Brandenburg, Germany). An array of seven hexagons was selected, and eight cycles for each hexagon were used for analysis. For each animal, the average amplitudes of the six hexagons around the optic nerve head were used for final analysis. The average a-wave or b-wave amplitudes of 6 to 8 animals were used for calculation.

### 4.6. Cell Culture and Transfection

Primary rat microglial cells and BV2 mouse microglial cells were cultured in DMEM medium containing 10% fetal bovine serum (FBS) (A4766801, Invitrogen) supplemented with 100 U/mL penicillin and 100 µg/mL streptomycin (Invitrogen) in a humidified incubator at 37 °C with 5% CO_2_. In 24-well cell culture format, 1 µL of 20 µM miRCURY LNA FITC labeled miR-124 mimic (479995-011, Qiagen), or miRCURY LNA FITC labeled control miRNA mimic (479903-001, Qiagen, sequence: 5′-GAUGGCAUUCGAUCAGUUCUA-3′), or FITC labeled miR-124 inhibitor (4102198-021, Qiagen) were transfected into cells using Lipofectamine 2000 Transfection Reagent (11668-027, Invitrogen) according to the manufacturer’s instructions.

### 4.7. Quantitative RT-PCR

Total RNA was prepared from rat retinae using TRIzol Reagent (Invitrogen) according to the manufacturer’s instructions. Gene expression was analyzed in a StepOne Plus Real-Time PCR system (Thermo Fisher Scientific, Dreieich, Germany) as previously described [21]. The catalog numbers of the primers are listed in Table 1.

### 4.8. Wound Healing Assay

After high glucose (4.5g/L) treatment for 24 h, BV2 cells were transfected with Opti-MEM^TM^ containing transfection complex (Lipofectamine 2000 addition with miR-124 mimic, or with control miRNA, or with miR-124 inhibitor) for another 24 h. Cells cultured in normal glucose medium (DMEM containing 1g/L glucose) and transfected with control miRNA were used as a control (NG-CTL). A scratch was made in the confluent cells, images were taken at time point 0 h and 24 h with the Leica DM IRB microscope, and at the endpoint, the cells were stained with Giemsa solution (T862.1 ROTH, Karlsruhe, Germany). Ten randomly selected wound distances from each image (four images/group) were measured with the Cell^F^ system (Olympus). The migration distance was calculated by subtracting the gap distance at 24 h from that at 0 h.

### 4.9. Immunocytochemistry

After transfection for 24 h, primary rat microglial cells were fixed with 4% PFA for 10 min and immunostained with primary antibodies against PU.1 (ab88082, Abcam, Berlin, Germany, 1:100) or FlOT1 (ab41927, Abcam, 1:100) at 4 °C overnight. Secondary antibody Alexa Fluor 555 donkey anti-mouse (A31570, Thermo Fisher Scientific) or Alexa Fluor 555 donkey anti-rabbit (A31572, Thermo Fisher Scientific) was applied at 1:200 dilution for one hour at room temperature. The nuclei were stained with DRAQ5^TM^ at 1:1000 dilution for 10 min at room temperature. After washing three times with PBS, the slides were mounted with fluorescence preserve VECTASHIELD^®^ HardSet^TM^ Antifade Mounting Medium (H-1400, Vector Laboratories, Eching, Germany). Images were taken with the Leica TCS SP8 confocal microscope.

### 4.10. Statistical Analysis

Data are presented as mean ± SD. Statistical analysis was performed using GraphPad Prism 8 (GraphPad Software, San Diego, CA, USA). Student *t*-test or one-way analysis of variance (ANOVA) with Tukey’s multiple comparisons test was used for comparison among the experimental groups. *p*-value < 0.05 was considered statistically significant.

## Figures and Tables

**Table 1:** List of primers used in this study.

Gene Name	Reference Number ^1^
*Arg1*	Rn00567522_m1
*Ccl2*	Rn00580555_m1
*Ccl3*	Rn01464736_g1
*Cd74*	Rn00565062_m1
*Flot1*	Rn00575535_m1
*Gapdh*	Rn99999916_s1
*Il-1ß*	Rn00580432_m1
*Il-10*	Rn00563409_m1
*Spi1*	Rn01513815_m1
*Tgf-ß1*	Rn00572010_m1
*Tnf-α*	Rn01525859_g1
*Vcam1*	Rn00563627_m1

^1^ All primers were from Thermo Fisher Scientific.

## Data Availability

Not applicable.

## Abbreviations

AC	acellular capillary
Arg1	arginase 1
CCL2	C-C motif chemokine ligand 2
CCL3	C-C motif chemokine ligand 3
CD74	major histocompatibility complex, class II invariant chain
C/EBP-α	CCAAT/enhancer-binding protein-α
CNS	central nervous system
DR	diabetic retinopathy
EAE	experimental autoimmune encephalomyelitis
ERG	electroretinography
Flot1	Flottilin-1
Gapdh	glyceraldehyde 3-phosphate dehydrogenase
GCL	ganglion cell layer
GFAP	glial fibrillary acidic protein
Iba1	Ionized calcium-binding adaptor molecule 1
IL-1β	interleukin-1β
IL-10	interleukin-10
ILM	inner limiting membrane
INL	inner nuclear layer
IPL	inner plexiform layer
miR-124	microRNA-124
miR-inh	microRNA-124 inhibitor
NPDR	non-proliferative diabetic retinopathy
NVU	neurovascular unit
OLM	outer limiting membrane
ONL	outer nuclear layer
OPL	outer plexiform layer
PFA	paraformaldehyde
PRL	photoreceptor layers
Spi1	Salmonella Pathogenicity Island 1 (gene name of PU.1)
STZ	streptozotocin
TNF-α	tumor necrosis factor-α
TGF-β1	transforming growth factor-β1
